# Cerebral Microbleeds with a Venous Connection on 3 Tesla Susceptibility-Weighted Imaging in Persons with Alzheimer’s Disease and Healthy Aging Controls

**DOI:** 10.3390/brainsci15080851

**Published:** 2025-08-10

**Authors:** Ulf Jensen-Kondering, Veronique Kuhn, Hannes Schacht, Alexander Neumann, Georg Royl, Peter Schramm

**Affiliations:** 1 Department of Neuroradiology, UKSH Campus Lübeck, Ratzeburger Allee 160, 23562 Lübeck, Germany; 2 Department of Neurology, UKSH Campus Lübeck, Ratzeburger Allee 160, 23562 Lübeck, Germany

**Keywords:** cerebral microbleeds, susceptibility-weighted imaging, cerebral amyloid angiopathy, Alzheimer’s disease

## Abstract

**Introduction**: It has been recently demonstrated that some cerebral microbleeds (CMBs) are connected to cerebral veins in patients with cerebral small vessel disease (CSVD) including cerebral amyloid angiopathy (CAA). We sought to demonstrate the presence of CMB at 3 Tesla using susceptibility-weighted imaging and speculated that it was more prevalent in persons with Alzheimer’s disease (AD), another amyloid-related disease, than in healthy ageing controls. **Material and Methods**: We included persons from the publicly available OASIS3-database. Persons were included if they had a structural MRI including a susceptibility-weighted sequence (SWI) and relevant clinical data. Two raters assessed the presence and location of CMBs and CMBs with a venous connection (CMB_ven_). **Results**: A total of 571 persons (AD, *n* = 140, healthy controls, *n* = 431) were included. In total, 367 CMBs were detected, encompassing 26/571 persons (4.5%) who had a total of 40/367 (10.9%) CMBs with a CMB_ven_, though there was no difference between persons with AD and healthy controls (AD 6.6%, healthy controls 7.4%, *p* = 0.773). Persons with CMB_ven_ had a higher total CMB load, were more likely female, displayed an APOE ε2/2 genotype and had antithrombotic treatment more often. Logistic regression revealed a higher number of CMB (OR (95% CI) = 1.351 (1.161–1.688), *p* < 0.0014) and a lower MoCA score (OR (95% CI) = 0.862 (0.762–0.982), *p* = 0.018), indicating a statistically significant association with the presence of CMB_ven_. **Discussion***:* CMB_ven_ are not an uncommon finding in persons with AD and healthy ageing controls. Our results highlight the potential venous contribution to CSVD. Histopathological studies will be needed to assess these further.

## 1. Introduction

The contribution of small cerebral veins to cerebrovascular small vessel disease (CSVD) is known but underexplored [1]. Venous collagenosis may lead to vessel occlusion and stenosis [2]. Amyloid deposition, not only in leptomeningeal and cortical arteries but also veins [3,4], is the hallmark of cerebral amyloid angiopathy (CAA). It has been recently demonstrated that a subset of cerebral microbleeds (CMBs), a neuroimaging feature of CSVD and CAA in particular [5], has a connection to veins (CMB_ven_) and may originate from the venous vasculature [6]. This demonstration was accomplished using high field strength (7 Tesla) and a dedicated imaging sequence and postprocessing algorithm (quantitative susceptibility mapping). The present study was conducted with two hypotheses, as follows: (1) The presence of CMB_ven_ can be demonstrated at a clinically available field strength (3 Tesla) and a widely available imaging sequence (susceptibility-weighted imaging, SWI) and (2) CMB_ven_ will be more prevalent in persons with Alzheimer’s disease (AD), another amyloid-driven pathology, than in healthy aging controls.

## 2. Materials and Methods

### 2.1. Database

Persons were included from the OASIS-3 database (https://sites.wustl.edu/oasisbrains/, last accessed 19 January 2024). The OASIS-3 database is a multimodal collection of clinical, laboratory and imaging data of >1000 participants accrued over the course of 15 years. It is managed by the Washington University Knight Alzheimer’s Disease Research Center and has been approved by the Institutional Review Board of Washington University School of Medicine. Persons gave their consent for the inclusion in the database and subsequent scientific use after anonymization [7]. Thus, the need to receive consent from individual persons was waived by the local ethics committee (Ethikkommision der Universität zu Lübeck, AZ 2024-403).

### 2.2. Participants

In a first instance, all persons with a diagnosis of AD according to the National Institute on Aging and Alzheimer’s Association (NIA-AA) criteria and the presence of a 3 Tesla structural MRI including a susceptibility-weighted sequence (SWI) were included. Then, all available non-demented persons with the presence of a 3 Tesla structural MRI including a susceptibility-weighted sequence (SWI) who did not fulfill the diagnostic criteria for Alzheimer’s disease were included. Persons were excluded if an intracranial tumor, a macrohemorrhage or severe image degradation due to artifacts was present.

### 2.3. Image Acquisition and Interpretation

All imaging was performed on a 3 Tesla SIEMENS Trio (Siemens Medical Solutions USA, Inc., Malvern, PA, USA) with a head coil and whole brain coverage. Sequence parameters were the following: TE = 200 ms, TR = 280 ms, flip angle = 15°, and slice thickness = 2 mm.

Two raters (UJK, board certified neuroradiologist with 15 years of experience, and VK, junior doctor with 1 year of experience) rated the number and presence of CMB according to the STRIVE criteria. For the purpose of this study CMBs in the frontal, parietal, temporal, occipital and insular lobes were categorized as lobar CMBs while basal ganglia, thalami and brain stem were categorized as deep CMBs. Cerebellar CMBs were counted as a separate region [8]. Mixed CMBs were defined as the presence of at least one CMB in a lobar and a deep region each.

A venous connection was rated as present if a venous vessel was detected in the direct vicinity, with no brain parenchyma between the vessel and the CMB. If there was disagreement, the rating of the first rater was used.

### 2.4. Clinical Parameters

Age, sex, race, apolipoprotein ε (APOE) genotype, arterial hypertension, a history of stroke or TIA, diabetes, hypercholesterinemia, antithrombotic treatment (any thrombocyte aggregation inhibitor or anticoagulation) and Montreal Cognitive Assessment (MoCA) score was recorded.

### 2.5. Statistics

R (version 4.4.1, R Foundation for Statistical Computing, Vienna, Austria) was used for statistical calculations. Mean, standard deviation, median, ranges and proportions are displayed for the variables as appropriate. Comparisons between groups were made with a *t*-test for normally distributed continuous variables, Mann–Whitney U test for non-normally distributed or ordinal scale variables, or a Chi-square test or Fisher’s exact test as appropriate for categorical variables. Interrater agreement was calculated using the intraclass correlation coefficient (ICC) for the total number of CMBs, while, for the assessment of the presence of CMB_ven_, Cohens’s κ was calculated. The total number of CMBs, gender, APOE genotype, MoCA score, age and antithrombotic therapy were included as variables in a logistic regression model. A Hosmer–Lemeshow test for the calculation of the goodness of fit of the model was performed. All tests were two sided and a *p*-value of <0.05 was considered statistically significant.

## 3. Results

### 3.1. Entire Cohort

A total of 571 persons could be included. Of these, 140 were judged to have AD, whereas 431 did not. Mean age was 69.3 ± 9.2 years, 59.4% (*n* = 339) were female and the majority (85.3%, *n* = 487) was white. Approximately half of the persons had a history of hypertension (45.5%) and hypercholesterinemia (46.1%) and were on antithrombotic treatment (50.9%). A minority of the persons had a history of TIA or stroke (4.4%) or diabetes (9.6%). Median MoCA score was 26 (interquartile range 23–28), Table 1.

### 3.2. Cerebral Microbleeds

A total of 367 CMBs in 116 individual persons were identified. ICC for the number of total CMBs was 0.98. The median number of CMBs per person was 0 with a range of 0 to 28. Most were located in lobar regions (75.5%), followed by deep (13.1%) and cerebellar (10.9%). A total of 15 persons had mixed CMBs.

### 3.3. AD vs. Healthy Controls

Persons in the AD group were significantly older (77 ± 6.7 vs. 67 ± 8.7 years, *p* < 0.001), less often female (49.3 vs. 62.6%, *p* = 0.004), displayed an APOE ε3/3 genotype more often (42.9 vs. 54.8%, *p* = 0.012) had a lower median MoCA score (21 vs. 27, *p* < 0.001), and were on antithrombotic treatment more often (64 vs. 47%, *p* < 0.001).

Persons in the AD group had less total CMBs per person (median 0, range 0–22 vs. 0, range 0–28, *p* = 0.034), more total deep CMBs (21.9 vs. 6.9%, *p* < 0.001), more deep CMBs per person (median 0, range 0–6 vs. 0, range 0–3, *p* < 0.001), a lower number of lobar CMBs (68.2 vs. 80.5%, *p* = 0.006), a lower number of cerebellar CMBs per person (median 0, range 0–3 vs. 0, range 0–13, *p* = 0.029) and a greater share of persons with mixed CMBs (6.4 vs. 1.4%, *p* = 0.001), Table 1.

### 3.4. Cerebral Microbleeds with a Venous Connection

**Figure 1:**
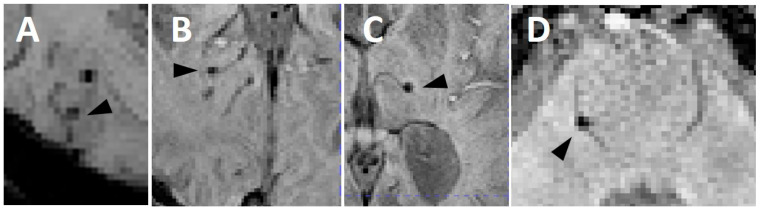
Examples of CMBs with a connection to veins (arrowheads) in the cortex (**A**,**B**), in the thalamus (**C**) and in the brain stem (**D**).

The total number of CMB_ven_ was 40 in 26 individual persons. Cohen’s κ for the presence of CMB_ven_ was 0.77. Most were located in lobar regions (93%), followed by deep (5%) and cerebellar (2%), Figure 1, Table 2.

Persons with CMB_ven_ were less often female (35 vs. 59.4%, *p* < 0.001); displayed an APOE ε 2/2 more frequently, although absolute numbers were very small (3.8 vs. 0.55%, *n* = 1 vs. *n* = 2, *p* = 0.016); were on antithrombotic treatment more often (73 vs. 50.2%, *p* = 0.005); had a lower median MoCA score (24 vs. 26, *p* = 0.014); and had more CMBs per person (median 3.5 vs. 0, *p* < 0.001), as shown in Table 3.

Only a higher number of CMB (OR (95% CI) = 1.351 (1.161–1.688), *p* < 0.0014) and a lower MoCA score (OR (95% CI) = 0.862 (0.762–0.982), *p* = 0.018) showed a statistically significant association with the presence of CMB_ven_ (Table 4). Goodness-of-fit calculation indicated a good model fit with χ^2^ = 8.467, *p* = 0.389.

## 4. Discussion

We have demonstrated that approximately 10% of the CMB found in patients with AD and healthy aging controls have a connection to cerebral veins on 3 Tesla using SWI, i.e., at a clinically available field strength and with a routinely employed MRI sequence. This number is in agreement with the results by Rotta and coworkers who demonstrated a connection of CMBs to small cerebral veins on QSM at 7 Tesla in patients with CSVD (CAA and hypertensive arteriopathy) and healthy controls in 14% of all CMBs [6]. In contrast to their work, we could not demonstrate a difference between persons with AD and healthy controls. A connection of CMBs with small cerebral veins has been previously described by Ayaz and coworkers in SWI at 1.5 Tesla in patients with mild cognitive impairment [9] as an adjunct finding. Their frequency however was not reported. A single case report of a patient with CAA-related inflammation also found most CMBs with a venous connection using SWI, though at a clinically unusual field strength (5 Tesla) [10].

It is widely accepted that amyloid plays a key role in the development of AD. Parenchymal amyloid depositions induce tau aggregations that lead to neuronal death and the typical clinical dementia [11]. While Aβ40 is the key player in CAA, Aβ42 is preferentially deposited in AD [12]. It is increasingly recognized that CAA and AD demonstrate a pathological overlap. This is exemplified by the fact that up to 80% of patients with AD exhibit neuropathological features of CAA [13]. Further, 30% of patients have CMBs [14].

Only a few studies have been dedicated to the contribution of veins to CSVD, a topic vastly underexplored compared with arteries. Collagenosis of small veins leads to wall thickening, stenosis and occlusion of small cerebral veins which may contribute to periventricular white matter lesions in patients with AD [2,15]. It is postulated that venous collagenosis [15] and venous Aβ deposition [16] impairs venous resistance and pulsatility [17], altering cerebral blood flow autoregulation [18] and reduces efflux of interstitial fluid, in turn impeding Aβ clearance and facilitating Aβ deposition [19]. In our study, the number of total CMBs and a lower MoCA score was associated with the presence of CMB_ven_. This could indicate that more severe tissue damage and advanced Aβ deposition could lead to more pronounced venous pathology.

In a mouse model, infarcts due to the occlusion of cortical venules were strikingly similar to infarcts due to the occlusion of arterioles [20]. However, several studies were unable to find a relationship between enlarged perivascular spaces and veins [21,22,23].

We cannot exclude the idea that these CMB constitute venous aneurysms [24] or that the assumed connection is indeed caused by mere spatial proximity and blooming artifacts [25] feigning an actual connection. Moreover, even if there is a contact point between CMB and a vein this association could still be by random and the CMB arterial. Further, additional features such as recently discovered blood-based biomarkers and other non-standard markers are not included in the OASIS-database and cannot be taken into account as a potential influence on the presence of CMB_ven_. Histopathological studies dedicated to the question of whether these CMBs are of arterial or venous origin, a subject that has been previously neglected [26], will shed light on this question.

## 5. Conclusions

CMB with a venous connection are not an uncommon finding. Our results put a focus on the potential venous side of CMBs and the contribution of veins in CSVD in general and amyloid-driven disease in particular. Histopathological studies will be needed to elucidate that relationship.

## Figures and Tables

**Table 1:** Demographics of the participants.

	Total (*n* = 571)	AD (*n* = 140)	No AD (*n* = 431)	*p*-Value
Age, mean ± SD	69.3 ± 9.2	77 ± 6.7 years	67 ± 8.7 years	<0.001 ^#^
Female sex, *n* (%)	339 (59.4)	69 (49.3)	270 (62.6)	0.004 ^§^
Race				
Asian, *n* (%)	3 (0.5)	0 (0)	3 (0.7)	1 *
Black, *n* (%)	80 (14)	14 (10)	66 (15.3)	0.11 ^§^
White, *n* (%)	487 (85.3)	126 (90)	361 (83.8)	0.094 ^§^
APOE				
ε2/2, *n* (%)	3 (0.5)	1 (0.7)	2 (0.46)	0.732 ^§^
ε2/3, *n* (%)	66 (11.5)	13 (9.3)	53 12.3)	0.324 ^§^
ε2/4, *n* (%)	12 (2.1)	1 (0.7)	11 (2.6)	0.185 ^§^
ε3/3, *n* (%)	296 (51.8)	60 (42.9)	236 (54.8)	0.012 ^§^
ε3/4, *n* (%)	166 (29)	56 (40)	110 (25.5)	0.001 ^§^
ε4/4, *n* (%)	25 (4.4)	8 (5.7)	17 (3.9)	0.380 ^§^
History of stroke/TIA, *n* (%)	25 (4.4)	10 (7.1)	15 (3.5)	0.065 ^§^
Arterial hypertension, *n* (%)	260 (45.5)	73 (52.1)	187 (43.4)	0.07 ^§^
Diabetes, *n* (%)	55 (9.6)	15 (10.7)	40 (9.3)	0.61 ^§^
Hypercholesterinemia, *n* (%)	263 (46.1)	66 (47.1)	197 (45.7)	0.767 ^§^
Antithrombotic treatment, *n* (%)	291 (50.9)	90 (64)	201 (47)	<0.001 ^§^
MoCA, median (IQR), range	26 (23, 28), 7–30	21 (17.5, 24), 7–30	27 (25, 28), 15–30	<0.001 ^$^

AD: Alzheimer’s disease; SD: Standard deviation; APOE: Apolipoprotein; TIA: Transient ischemic attack; MoCA: Montreal cognitive assessment; IQR: Interquartile range. ^#^: *t*-test; ^§^: χ^2^-test; *: Fisher’s exact test; ^$^: Mann–Whitney U test.

**Table 2:** Results for the cerebral microbleeds (CMBs).

	Total (*n* = 571)	AD (*n* = 140)	No AD (*n* = 431)	*p*-Value
Persons with any CMB, *n* (%)	116 (20.3)	36 (25.7)	80 (18.6)	0.067 ^§^
Total CMB, *n*	367	151	216	NA
CMB per person, median (IQR), range	0 (0, 0), 0–28	0 (0, 1), 0–22	0 (0, 0), 0–28	0.034 ^$^
Deep CMB total, *n* (%)	48 (13.1)	33 (21.9)	15 (6.9)	<0.001 ^§^
Deep CMB per person, median (IQR), range	0 (0, 0), 0–6	0 (0, 0), 0–6	0 (0, 0), 0–3	<0.001 ^$^
Lobar CMB total, *n* (%)	277 (75.5)	103 (68.2)	174 (80.5)	0.006 ^§^
Lobar CMB per person, median (IQR), range	0 (0, 0), 0–28	0 (0, 0), 0–16	0 (0, 0), 0–28	0.124 ^$^
Persons with mixed CMB, *n* (%)	15 (2.6)	9 (6.4)	6 (1.4)	0.001 ^§^
Cerebellar CMB total, *n* (%)	40 (10.9)	15 (9.9)	25 (11.6)	0.084 ^§^
Cerebellar CMB per person, median (IQR), range	0 (0, 0), 0–13	0 (0, 0), 0–3	0 (0, 0), 0–13	0.029 ^$^
Persons with any CMB_ven_, *n* (%)	26 (4.5)	10 (6.6)	16 (7.4)	0.773 ^§^
Total CMB_ven_, *n* (%)	40 (10.9)	12 (7.9)	28 (12.9)	0.403 ^§^
Deep CMB_ven_, *n* (%)	2 (0.5)	2 (1.3)	0 (0)	0.059 *
Deep CMB_ven_ per person, median (IQR), range	0 (0, 0), 0–1	0 (0, 0), 0–1	0 (0, 0), 0	0.013 ^$^
Lobar CMB_ven_, *n* (%)	37 (10.1)	10 (6.6)	27 (12.5)	0.713 ^§^
Lobar CMB_ven_ per person, median (IQR), range	0 (0, 0), 0–4	0 (0, 0), 0–2	0 (0, 0), 0–4	0.181 ^$^
Cerebellar CMB_ven_, *n* (%)	1 (0.3)	0 (0)	1 (0.5)	1 *
Cerebellar CMB_ven_ per person, median (IQR), range	0 (0, 0), 0–1	0 (0, 0), 0	0 (0, 0), 0–1	0.571 ^$^

AD: Alzheimer’s disease; IQR: Interquartile range. ^§^: χ^2^-test; *: Fisher’s exact test; ^$^: Mann–Whitney U test, NA: not applicable.

**Table 3:** Results for the cerebral microbleeds (CMBs) with and without a venous connection.

	CMB_ven_ Present (*n* = 26)	CMB_ven_ Absent (*n* = 545)	*p*-Value
Age, mean ± SD	72.3 ± 8.8 years	68.9 ± 9.2	0.069 ^#^
Female sex, *n* (%)	9 (35)	324 (59.4)	<0.001 ^§^
Race			
Asian, *n* (%)	0 (0)	3 (0.5)	1 *
Black, *n* (%)	2 (7.7)	76 (13.9)	0.364 ^§^
White, *n* (%)	24 (92.3)	465 (85.3)	0.320 ^§^
APOE			
ε2/2, *n* (%)	1 (3.8)	2 (0.55)	0.016 ^§^
ε2/3, *n* (%)	2 (7.7)	63 (11.6)	0.544 ^§^
ε2/4, *n* (%)	0 (0)	12 (2.2)	1 *
ε3/3, *n* (%)	16 (62)	288 (52.8)	0.385 ^§^
ε3/4, *n* (%)	5 (19.2)	153 (28)	0.324 ^§^
ε4/4, *n* (%)	2 (7.7)	24 (4.4)	0.431 ^§^
History of stroke/TIA, *n* (%)	2 (7.7)	23 (4.2)	0.397 ^§^
Arterial hypertension, *n* (%)	10 (38.4)	248 (45.5)	0.480 ^§^
Diabetes, *n* (%)	3 (11.5)	52 (9.5)	0.735 ^§^
Hypercholesterinemia, *n* (%)	9 (34.6)	248 (45.5)	0.275 ^§^
Antithrombotic treatment, *n* (%)	19 (73)	274 (50.2)	0.005 ^§^
MoCA, median (IQR), range	24 (20, 25.75), 8–28	26 (24, 28), 7–30	0.014 ^$^
CMB per person, median (IQR), range	3.5 (2, 5), 1–28	0 (0, 0), 0–21	<0.001 ^$^

AD: Alzheimer’s disease; SD: Standard deviation; APOE: Apolipoprotein; TIA: Transient ischemic attack; MoCA: Montreal cognitive assessment; IQR: Interquartile range. ^#^: *t*-test; ^§^: χ^2^-test; *: Fisher’s exact test; ^$^: Mann–Whitney U test.

**Table 4:** Logistic regression analysis of factors associated with the presence of CMB_ven_. Odds ratios (OR), 95% confidence intervals (CI) and *p*-values are reported. * indicates statistical significance (*p* < 0.05).

Variable	OR (95% CI)	*p*-Value
Total CMB number	1.351 (1.161–1.688)	<0.0014 *
Gender	1.427 (0.392–5.419)	0.585
APOE ε genotype	1.558 (0.002–92.812)	0.874
MoCA score	0.862 (0.762–0.982)	0.018 *
Age	0.968 (0.899–1.045)	0.397
Antithrombotic treatment	0.916 (0.246–3.470)	0.894

## Data Availability

Data are contained within the article. These data were derived from the following resources available in the public domain: https://sites.wustl.edu/oasisbrains/, https://sites.wustl.edu/oasisbrains/ last accessed 19 January 2024. Further material is available from the corresponding author upon reasonable request.

## Abbreviations

The following abbreviations are used in this manuscript:

AD	Alzheimer’s disease
APOE	Apoliptoprotein E
CAA	Cerebral amyloid angiopathy
CMB	Cerebral microbleed
CMB_ven_	Cerebral microbleeds with a venous connection
CVSD	Cerebrovascular small vessel disease
MoCA	Montreal cognitive assessment
SWI	Susceptibility-weighted imaging
